# Radio Frequency Database Construction and Modulation Recognition in Wireless Sensor Networks

**DOI:** 10.3390/s22155715

**Published:** 2022-07-30

**Authors:** Kun Liu, Xin Xiang, Liyan Yin

**Affiliations:** College of Astronautics Engineering, Air Force Engineering University, Xi’an 710038, China; xxisdn2002@163.com (X.X.); yinliyanafeu@163.com (L.Y.)

**Keywords:** wireless sensor networks, modulation recognition, radio frequency database, multipath fading channels

## Abstract

Current modulation recognition methods in wireless sensor networks rely too much on simulation datasets. Its practical application effect cannot reach the expected results. To address this issue, in this paper we collect a large amount of real-world wireless signal data based on the software radio device USRP 2920. We then propose a real radio frequency (RF) database architecture and preprocessing operators to manage real-world wireless signal data, conduct signal preprocessing, and export the dataset. Based on different feature datasets derived from the RF database, we propose a multidimensional feature hybrid network (MFHN), which is used to identify unknown signals by analyzing different kinds of signal features. Further, we improve MFHN and design a multifeatured joint migration network (MJMN) to identify small-sample targets. The experimental results show that the recognition rates for unknown target signals of the MFHN and MJMN are 82.7% and 93.2%, respectively. The proposed methods improve the recognition performance in the single node of wireless sensor networks in complex electromagnetic environments, which provides reference for subsequent decision fusion.

## 1. Introduction

Multiple monitoring nodes in wireless sensor networks can perform detailed signal feature analysis, thereby improving the performance of signal modulation recognition. As the basis for multi-node decision fusion, the accuracy of recognition results in a single monitoring node is critical [1]. Due to the constraints of noncollaborative communication and the interference of multipath fading channels, the features of the transmission signals change dramatically, affecting the signal modulation recognition results in single monitoring node.

At present, there are two categories to improve recognition performance: likelihood-based methods and feature-based methods. The former [2,3] has high recognition accuracy, but needs more prior information and complex calculation. The latter [4,5] requires less prior information and simple calculation and shows better recognition performance in practical application. In a real communication scenario, analyzing the mechanism of signal characteristic changes is a key step for accurate modulation recognition. Sufficient real acquisition signals are the premise of feature-based methods. Admittedly, traditional analysis methods can no longer meet the accuracy requirements of large-scale signal data processing. It is difficult to analyze different kinds of signal characteristics. As a result, deep learning is applied in the signal processing field with powerful optimization and learning abilities, such as signal recognition [6], blind equalization [7], and spectrum sensing [8].

The deep learning methods can extract more reliable characteristics from the signals in wireless sensor networks, and then achieve the purpose of signal analysis and recognition. However, most of the intelligent modulation recognition methods that are widely considered at present are based on simulated signal data or a small number of actually collected signal data. In the literature, a signal simulation dataset called RML2016.10a has been designed for automatic modulation [9], which is the basis of many other modulation recognition methods. The literature [10] expanded the dataset RML2016.10a and improved its performance. In addition, a simulation dataset is proposed in the literature [11] based on MATLAB, and eye maps and vector maps are extracted for modulation recognition. However, the above methods only work well when the signal source parameters to be identified are consistent with those of the training dataset. These recognition rates decrease severely in the real communication environment. According to the above-simulated datasets, a series of methods based on advanced signal features, such as time-frequency maps [12], constellation maps [13], waveform maps [14], instantaneous characteristics, and high-order cumulants [15], have been derived. However, these methods are essentially dependent on simulated datasets, and the recognition rates under noncooperative communication conditions cannot meet expectations. Therefore, some scholars have started to focus on real acquisition signal datasets. The E4438 signal source is used to generate modulation signals and acquire real datasets from an ESMD receiver for research in the literature [16]. It collects signals in real communication scenes and produces datasets that can be better used in practice. However, the simple channel condition makes it impossible to meet the real communication requirements. Reference [17] considered that if there are no common waveform datasets collected in a rich and diverse wireless communication environment, all the methods on radio recognition can achieve satisfactory results. The paper constructed a large-scale real signal dataset in a variety of scenarios through USRP, but it did not propose an effective network framework to learn signal features. Moreover, the data captured by the reconnaissance receiver may contain many other signals. The diversity of the modulation modes and the interweaving of different signals make the captured target signals unable to be directly used for recognition, and pretreatment operations such as signal sorting must be carried out for better effects [18].

Modulation recognition of real acquisition signals is currently difficult to achieve. There are substantial differences between signal recognition and common image recognition in nature. The image features in the training and test sets differ slightly from those in actual applications. The trained model can be used directly [19]. Conversely, signal recognition receives interference from many factors. For example, receiver differences, channel interference, and noise all affect the signal features. There are considerable differences between the real acquisition signal data to be identified and the simulation data used to train the network model. Therefore, the neural network framework trained through the simulation data cannot obtain a satisfactory recognition result under complex and variable wireless channels, which has less application value for radio communication in real environments [20].

To achieve practical modulation recognition in wireless sensor networks, a large number of real signals must be collected under different communication environments and channel conditions. However, the target signal is interfered with by other signals in the acquisition process and cannot be directly used as a sample for training. Original signals must be obtained through signal preprocessing before they can be used for neural network learning and training. Signal resampling is necessary for subsequent training. Moreover, signal data resources are abundant, and fine signal recognition requires considerable manual analysis. Therefore, traditional file storage methods cannot meet the needs of large-scale signal analysis. However, we manage the pre-processed signal directly and export the dataset through the database. The exported dataset can be used directly for training the neural network, avoiding complex data analysis. It is urgent to build a database equipped with a signal preprocessing operator to achieve signal data resource integration.

The purpose of our method is to provide a single node modulation recognition method in wireless sensor networks, and its recognition results can be used for decision fusion in multi-sensor node modulation recognition.

To improve the reliability of modulation recognition methods and the analysis ability of real acquisition signals, we construct an RF database and preprocessing operators for intelligent signal processing. It can perform functions of signal storage and invocation, signal data preprocessing, and signal feature extraction. Furthermore, we propose MHN and MJMN frameworks to validate the database capabilities, dealing with issues about how to train the signal features of different dimensions and identify small-sample targets. The main contributions of this paper are as follows:

1. We build a specialized RF database for wireless signal processing. It can perform the signal filtering, resampling, and feature extraction with a signal preprocessing operator, which simplifies the front-end signal processing operation and realizes large-scale real signal management.
2. We can obtain different types of datasets from the RF database according to different signal processing purposes in wireless sensor networks, which improve the practical application performance of the training model.
3. We propose a multidimensional feature hybrid network framework, construct subnetwork modules according to the different dimensional signal feature datasets, and explore the mapping relationship between different features and signal modulation modes.
4. We propose a multifeatured joint migration network framework to identify uncommon samples. Considering the lack of data for training in real communication scenarios, we design MJMN from three aspects: dataset generation, feature extraction, and network framework construction.

The remaining sections are arranged as follows: In Section 2, the signal acquisition process and the preprocessing operator is described. In Section 3, we introduce the structure and the functions of an RF database. In Section 4, we use the real signal dataset exported from the database to train the neural network. The proposed network model has the ability of multi-feature extraction and small sample signal recognition. In Section 5, the performance of our methods is analyzed and compared with the experimental results. Finally, we summarize the proposed method in Section 6.

## 2. Signal Acquisition and Preprocessing Operator

Collecting real signal data and designing a signal preprocessing operator are the basis of an RF database for data analysis and management. The former is used to provide data sources and the latter is used to provide pure signal data for neural network training through signal filtering, resampling, and feature extraction.

### 2.1. Signal Acquisition

**Figure 1:**
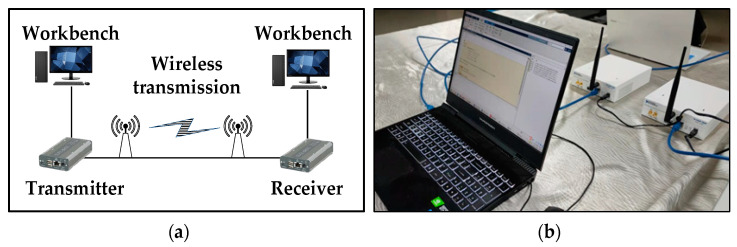
RF acquisition system. (**a**) Hardware connection; (**b**) USRP 2920.

The connection relationship and hardware configuration of the signal acquisition device are shown in Figure 1. We use two USRP 2920 as RF transmitters and receivers, in which RF and IF signal conversion is completed. The RF center frequency is 400 MHz, and the IF frequency is 70 MHz. USRP connects to the workbench through the Ethernet to complete baseband signal processing. In the experiments involved in this paper, the symbol rate of the transmitted signal is 400 kps, and the sampling rate is 800 kHz. The modulation modes are set to 2FSK, 16QAM, 64QAM, BPSK, MSK, QPSK, and 2ASK, and root-lift cosine shaping with a roll-off factor of 0.25. For each modulation mode, we collect 1 s at the receiver, which is 800 K sampling points. The original sample signal file collected in the workbench is saved in the CSV format.

### 2.2. Signal Preprocessing

The objective of signal preprocessing is a single original sampling signal file, which is realized by the preprocessing operator embedded in the RF database. The operator is designed and implemented based on MATLAB 2021b, including loading analogy channels and noise and standardized operation. The signal preprocessing result file is directly generated through the input data via the operator. We choose the Rayleigh multipath fading channel model described in the literature [21], and the communication and baseband complex discrete signal passing through the channel can be expressed as:

(1) x(n)=s(n)∗h(n)+w(n)

where *s*(*n*) represents the input signal, *h*(*n*) represents the channel coefficient of the 5-path channel; the path delays and variances are shown in Table 1. *w*(*n*) represents Gaussian additive white noise (AWGN).

Next, we normalize the signal with zero mean and variance. The influence of the signal mean and power on feature extraction is eliminated and can be expressed as:

(2) $$x^{\prime }(n)=\frac{x(n)-\mu }{\sigma }$$

where *μ* represents the mean of the signal and *σ* represents the standard deviation in the signal. The preprocessed signals are saved in the CSV format.

### 2.3. Feature Extraction

The objective of feature extraction is the signal dataset exported from the database. It is also realized by the preprocessing operator. The purpose of feature extraction is to extract the features of each sample in the export signal dataset and produce one or more feature datasets, which can be implemented by the pre-set feature extraction operator in the platform. In this paper, we use orthogonal sampling waveform and high-order cumulative and time-frequency maps to form three new feature datasets. All signal datasets and three corresponding feature datasets are applied to train the network framework. 

#### 2.3.1. Higher-Order Cumulant

High-order cumulants are embodied in the form of point eigenvalues [22]. It is one of the features commonly used in modulation recognition. It can weaken the influence of noise on the signal. The higher-order moment of the signal can be defined as:

(3) $$M_{pq}=E[x^{p-q}(n)x^{*}{\left(n\right)}^{q}]$$

where * represents the conjugate of the complex signal sequence and *q* is the number of conjugate sequences. For the stationary complex signal, *x*(*n*), with zero mean, the expression of its second-order cumulants is as follows [22]:

(4) $$C_{20}=Cum(x,x)=M_{20}$$

(5) $$C_{21}=Cum(x,x^{*})=M_{21}$$

Its fourth-order cumulants are as follows:

(6) $$C_{40}=Cum(x,x,x,x)=M_{40}-3M_{20}{}^{2}$$

(7) $$C_{41}=Cum(x,x,x,x^{*})=M_{41}-3M_{21}M_{20}$$

(8) $$C_{42}=Cum(x,x,x^{*},x^{*})=M_{42}-M_{20}{}^{2}-2M_{21}{}^{2}$$

Its sixth-order cumulants are as follows:

(9) $$C_{60}=Cum(x,x,x,x,x,x)=M_{60}-15M_{40}M_{20}+30M_{20}{}^{3}$$

(10) $$C_{61}=Cum(x,x,x,x,x,x^{*})=M_{61}-5M_{40}M_{21}-10M_{20}M_{41}+30M_{21}M_{20}{}^{2}$$

(11) $$C_{63}=Cum(x,x,x,x^{*},x^{*},x^{*})=M_{63}-6M_{41}M_{20}-9M_{21}M_{42}+18M_{21}M_{20}{}^{2}+12M_{21}{}^{3}$$

#### 2.3.2. Quadrature Sampling Waveform

The signal orthogonal sampling waveform is the data segment of the signal sampling point after preprocessing. Because the data do not undergo any feature extraction and have no information loss, they contain rich original signal features and can be directly used for deep-seated feature extraction.

#### 2.3.3. Signal Time-Frequency Maps

**Figure 2:**
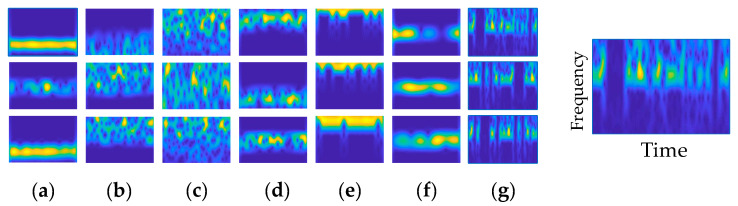
Time-frequency maps, where the horizontal axis represents time and the vertical axis represents frequency. (**a**) 2FSK; (**b**) 16QAM; (**c**) 64QAM; (**d**) BPSK; (**e**) MSK; (**f**) QPSK; (**g**) 2ASK.

The signal time-frequency map reflects the time and frequency characteristics locally [23]. It can preserve the original characteristics of the signal under the condition of multipath fading channels. In this paper, the time-frequency map is extracted by short-time Fourier transform (STFT), which can better reflect the time-frequency characteristics of the signal.

(12) $$\mathrm{STFT}(t,w)=\int_{-\infty }^{+\infty }x(\tau )g(\tau -t)e^{-jwt}d\tau$$

where *x*(*t*) represents the modulation signal to be identified and *g*(*t*) represents the window function; the time-frequency relationship of some preprocessed signals is shown in Figure 2.

## 3. Radio Frequency Database Construction

**Figure 3:**
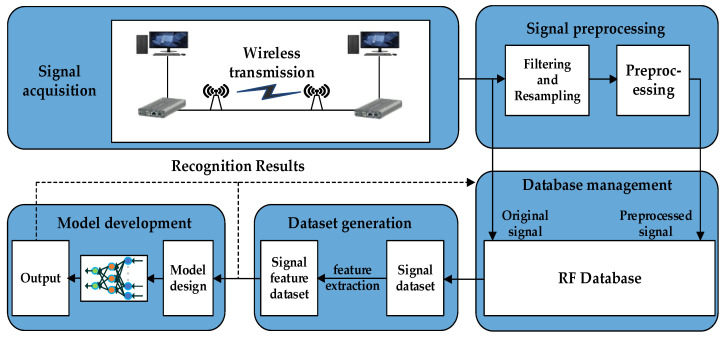
The formation process of the RF database.

The formation process of the RF database is shown in Figure 3, including five steps: RF signal acquisition, signal preprocessing, database management, dataset generation, and model development. The core function is to establish a real signal data source for intelligent signal processing. 

### 3.1. The Function of RF Database

1. Signal Acquisition

Signal acquisition is the basis of the RF database, which is used to store the collected real signal data and transmit it to the database.

2. Signal Preprocessing

Signal preprocessing achieves filtering, resampling, and preprocessing of the original signal and uploads the preprocessed signal to the database for storage and management.

3. Database Management

Database management is to store and manage the real acquisition signals and preprocessed signals, through which we can export different types of datasets according to the signal processing purpose.

4. Dataset Generation

Dataset generation is to select data samples from the database according to the index items. We intercept the fixed-length and labeled sampling point data segments to generate the signal dataset, and the signal feature dataset is also obtained through the feature extraction preprocessing operator.

5. Model Development

Model development completes the model training, development, and practical application deployment of neural networks according to different signal processing tasks. Furthermore, each training result and corresponding dataset is uploaded to the database for storage and use.

### 3.2. RF Database Design

The storage items of the database include the original sampling signal file, the preprocessed signal file, the signal dataset, the feature dataset, the training result, the application dataset, the application feature dataset, and the application result. Details of each storage item are shown in Table 2. In the item “Storage Format”, “Records in database” means sampling signal files obtained through USRP 2920, which represents records stored in the database. “Records” means preprocessed signal files obtained through sampling signal files, which represents records for the dataset. “Records in database” is the same as “Records” in format. “Samples” means samples in the signal dataset, which is obtained through intercepting the fixed long sampling points from “Records”.

**Figure 4:**
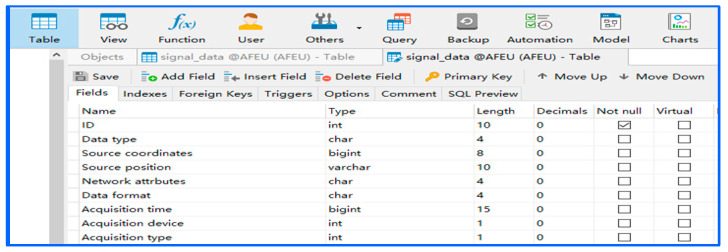
Index item information.

Each original sampling signal file is uploaded to the database as a record with index items such as source attributes, acquisition parameters, and basic signal characteristics, covering all basic signal information. The index item configuration is shown in Table 3. According to different index items, we filter different original sampling signal records or preprocessed signal records in the database and export subsequent datasets. The index item information is shown in Figure 4.

### 3.3. Dataset Generation from RF Database

**Figure 5:**
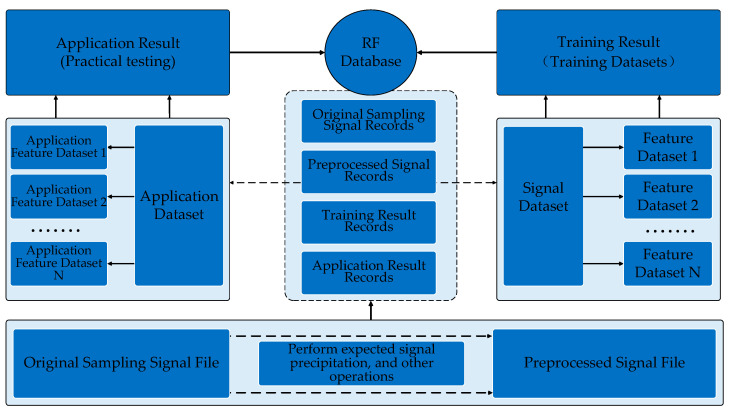
Database framework.

As shown in Figure 5, the original sampling signal file is preprocessed to obtain a new preprocessed signal file. The two kinds of files are uploaded to the database together and stored in the form of data records. For the preprocessed signal records, we select different index items to filter the data records for training according to the recognition purpose and intercept the fixed long sampling points from them. A preprocessing signal record can intercept one or more sampling point data segments.

**Figure 6:**
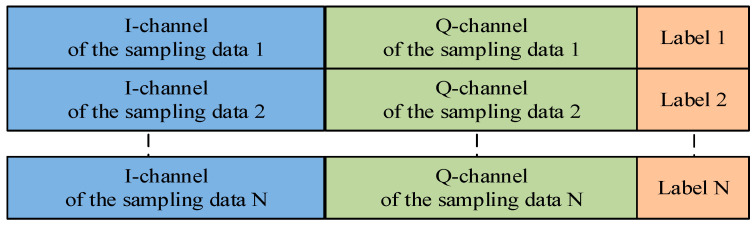
Dataset structure.

We add labels to each data segment to form dataset samples. Multiple fixed-length dataset samples containing different labels are combined to form the dataset for training. The structure of the datasets exported from the database is shown in Figure 6 and are used for verifying the performance of our methods.

Based on the existing signal datasets, the feature extraction operator designed is used to extract the features of each sample. We then obtain the labeled feature datasets with the same number of samples. Different features can form multiple feature datasets. Considering the different identification requirements, one or more of the signal datasets and data feature sets can be selected to complete the network framework training. At the same time, the training results are saved to the database as records for subsequent use.

At present, the parameters of the training set and test set in most methods based on open datasets are identical, and the recognition effect is satisfactory for identifying signals with the same parameters. However, when identifying signals with inconsistent parameters, the recognition performance is greatly reduced, which cannot meet the practical application requirements.

To analyze the practicability of the method, we design an application dataset and application feature datasets whose parameters are completely different from those of the signal datasets. These two kinds of datasets can be regarded as the unknown signal intercepted in the real communication environment and are used to assess the practical application performance of the trained models.

## 4. Radio Frequency Database Construction

Based on the real signal dataset exported from the database, we design MFHN for training different dimensional signal features. Furthermore, we designed an MJMN according to the weight parameters of MFHN. It has the ability of multi-feature extraction and small sample signal recognition.

### 4.1. Multidimensional Feature Hybrid Network Framework

Features play an important role in signal recognition. They are the wireless signal fingerprints and are the basis for distinguishing different modulated signals. Signal features cover the time domain, frequency domain, time-frequency domain, wavelet domain, and other transformation domains. We classify the signal features into three categories according to their dimensions: point features, line features, and surface features. Point features are represented as a single eigenvalue, including the instantaneous frequency, amplitude, phase, and cumulative frequency. Line features are expressed as one-dimensional (1D) vectors, including the probability density function, power spectrum, amplitude spectrum, and original waveform. Area features are represented as two-dimensional (2D) matrix graphs, including signal constellation, time-frequency map, eye map, and other signal vector map features.

**Figure 7:**
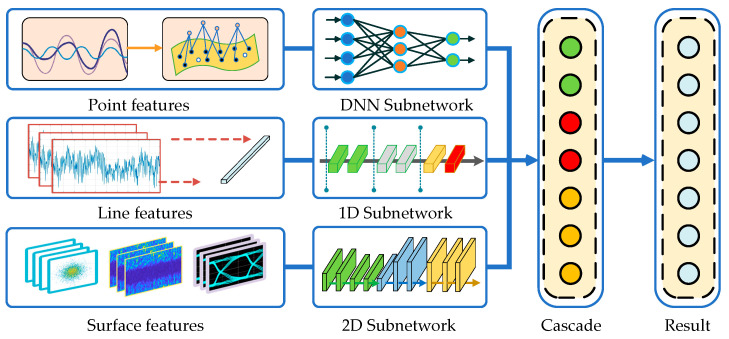
MFHN recognition framework.

Aiming at different dimensional features, we propose a MFHN recognition framework, which supports parallel input for signal point, line, and surface features. The proposed framework consists of a deep neural subnetwork module, a 1D subnetwork module, and a 2D subnetwork module, and its structure is shown in Figure 7.

Due to the different types of input signal features, the subnetwork parameters in MFHN are configurable. In this paper, we select high-order cumulative values as signal point features, original sample waveforms as signal line features, and time-frequency maps as signal surface features to configure subnetwork parameters of different dimensions.

For signal point features, we design a deep neural network (DNN), which is composed of a long-short-term memory neural network (LSTM) unit and five fully connected layers. It is used to explore the nonlinear relationship between the point features and the modulation modes. The ReLu is selected as the activation function. The network parameters are shown in Table 4. The network input is eight higher-order cumulative values obtained by the signal preprocessing operator.

For signal line features, we design a 1D convolution neural network (1D-CNN) structure. The convolution kernel takes a one-dimensional structure, which can extract different types of signal waveform features sufficiently. Additionally, the data redundancy is reduced by setting the step size. Due to the one-dimensional characteristics of the input signal, a bidirectional LSTM layer is introduced to prevent long-sequence gradient explosion during training. The network parameters are shown in Table 5. The network input is an orthogonal sampling signal with dimensions of 512 × 1.

For signal surface features, we design a two-dimensional convolution neural network (2D-CNN). The network is composed of four 2D convolution layers. The convolution layer is used to learn the features of the input feature map, which reduces the complex manual feature extraction steps and achieves high-level feature extraction through low-level feature combinations. The network parameters are shown in Table 6. The network input is a signal time-frequency map with dimensions of 128 × 128 × 3.

Through a feature cascade, the output of three subneural networks is combined, and signal recognition is achieved by using softmax activation. The parallel input of MFHN are eight higher-order cumulative values with dimensions of 8 × 1, an orthogonal sampling signal with dimensions of 512 × 1, and a signal time-frequency map with dimensions of 128 × 128 × 3. The output of MFHN is a row vector with dimensions of 7 × 1. Furthermore, the initial learning rate, batch size, and the epoch are set to 0.001, 256, and 1000.

### 4.2. Multifeatured Joint Migration Network Framework

**Figure 8:**
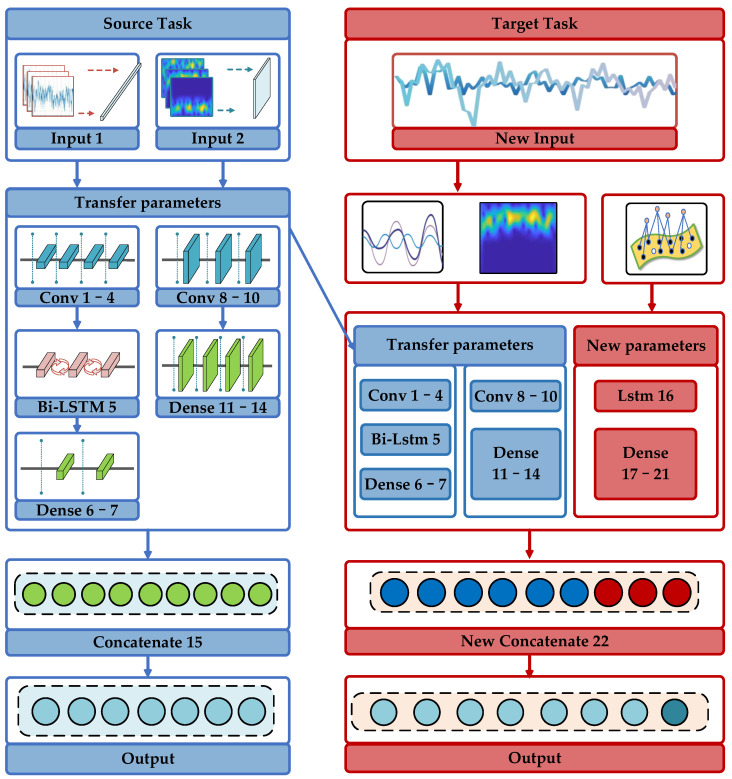
MJMN recognition framework.

In the real communication environment, the number of some original acquisition signal files is too small to support the target network training. For this reason, we propose a MJMN recognition framework based on the MFHN, which can optimize the existing training models with new data and new modulation modes. The structure of the new network is shown in Figure 8.

This network framework improves the learning ability for small sample objects from three aspects: dataset generation, multifeatured extraction, and network structure design. For dataset generation, we classify the original acquisition signal records into two categories: rich original signal records and a small number of target signal records. Only one sample point data segment is intercepted in the former, while multiple sample point data segments are intercepted in the latter. In this way, the number of samples of the target dataset to be tested is increased. For feature extraction, we extract the high-order cumulant and time-frequency maps of signals from each dataset sample and form corresponding feature datasets and then use a hybrid network structure to train it; in this way, the number of target datasets to be tested is increased. For structure design, we draw lessons from the idea of migration learning. First, we use the data-rich signal samples to train the 1D-CNN and 2D-CNN and migrate their network structure and weight parameters to the target network to be identified. The weights of the 1D-CNN and 2D-CNN are then fine-tuned through learning the target dataset. At the same time, the DNN is introduced to extract point features individually. Finally, the classification network framework is built by a cascade of features.

## 5. Analysis of Experimental Results

To verify the validity and practicability of the proposed framework, we make the experimental comparison and analysis from four aspects. First, we analyze the performance of the MFHN when identifying signals with consistent and inconsistent parameters. Second, we select different input features to analyze the impact of input features on the network performance. The recognition rates of the proposed network and other networks are then compared. Finally, the performances of the MFHN and MJMN are compared to verify the latter’s ability to recognize signals with fewer samples.

The datasets are derived from the RF database. The signal dataset 1 for training contains modulated signals with different carrier frequencies collected by USRP2920. The modulation modes are 2FSK, 16QAM, 64QAM, BPSK, MSK, QPSK, and 2ASK, and each modulation mode corresponds to 2000 samples. The acquisition rate is 800 ksp/s. The acquisition form is complex sampling. The sampling precision is int16, the sampling time is 1 s, and the total number of sample points is 800 k. Each data record intercepts a dataset sample with a label-free sample length of 512 × 1. The ratio of the test set to the training set is 2:8.

Application dataset 2 used for the actual application performance test contains modulated signals collected by USRP2920, whose parameters are inconsistent with the signal dataset for training. The modulation modes are 2FSK, 16QAM, 64QAM, BPSK, MSK, QPSK, and 2ASK, and each modulation mode corresponds to 200 samples. Each data record produces one sample, and the length of the sample is 512 × 1.

Target dataset 3 with fewer samples to be tested for signal recognition contains modulated signals collected by USRP2920, whose parameters are inconsistent with the signal dataset and application dataset. The modulation modes are 2FSK, 16QAM, 64QAM, BPSK, MSK, QPSK, and 2ASK, and each modulation mode corresponds to 10 samples.

### 5.1. Recognition Result Confusion Matrices

**Figure 9:**
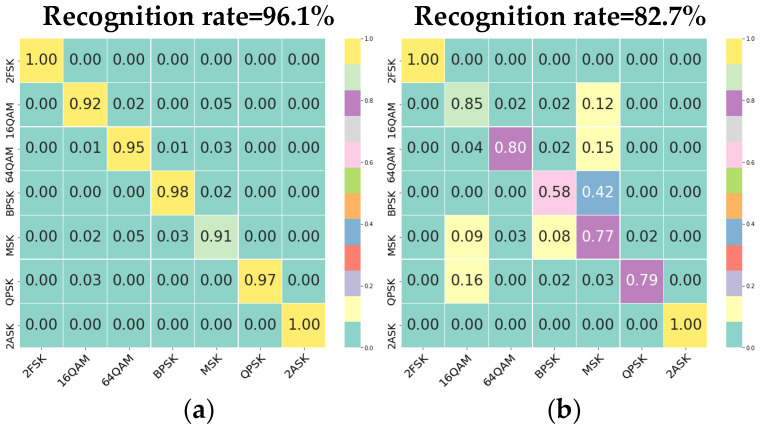
Multidimensional hybrid network confusion matrices. (**a**) This is the training result of dataset 1 in which the signal parameters to be recognized are consistent with those of the training set. (**b**) This is the training result of dataset 2 in which the signal parameters to be recognized are inconsistent with those of the training set.

Figure 9 shows the confusion matrices obtained through our method based on dataset 1 when we use higher-order cumulative values, original sampling waveforms, and signal time-frequency maps as input. The recognition results of the MFHN in signal dataset 1 are shown in Figure 9a. The vertical axis represents the real modulation mode, and the value corresponding to the horizontal axis represents the probability of recognizing the real modulation mode. When the training set and the test set parameters are consistent, the recognition performance of the algorithm is good, and the overall recognition rate reaches 96.1%. The recognition rate of 2FSK and 2ASK is 100%. However, the recognition results in Figure 9b are different. When the test set changes to application dataset 2 and the weight of the network framework remains unchanged, that is, the signal parameters to be recognized are inconsistent with those of the training set, the recognition performance of the algorithm decreases to 82.7%. At this time, the network framework only has a high recognition rate for 2FSK and 2ASK and a low recognition rate for modulation modes such as BPSK and MSK.

### 5.2. Comparison of Experimental Results with Different Input Features

**Figure 10:**
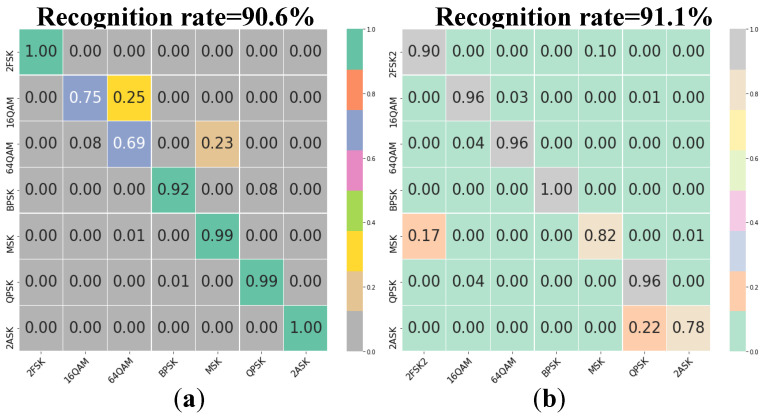
Experimental results on signal dataset 1 in which the signal parameters to be recognized are consistent with those of the training set. (**a**) Original sampling waveform. (**b**) Time-frequency map.

Figure 10 shows the confusion matrices obtained through 1D-CNN or 2D-CNN based on dataset 1 when we use one of original sampling waveforms and time-frequency maps as input. As shown in Figure 10, we select the different feature input to compare with our recognition results when the parameters of the training set and the test set are identical. The overall recognition rate of the method based on the original sampling waveform is 90.6%, and that of the method based on the time-frequency map is 91.1%. Both are lower than the recognition rates obtained in this paper.

**Figure 11:**
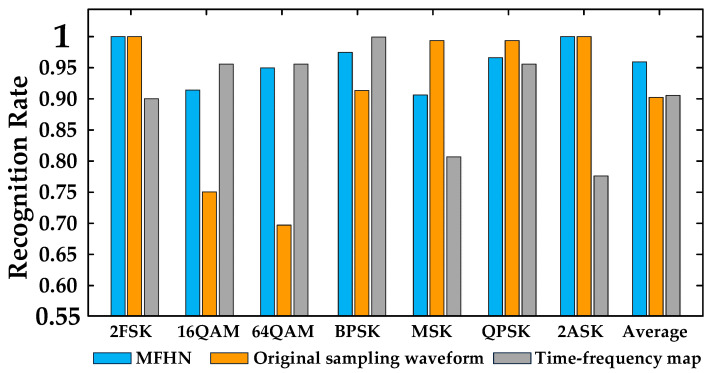
Modulation recognition based on signal dataset 1.

Figure 11 shows the recognition rate for a single modulation mode through MFHN (mixed features), 1D-CNN (original sampling waveforms), and 2D-CNN (signal time-frequency maps) based on dataset 1. For 2FSK, MSK, QPSK, and 2ASK modulation, the recognition rate of the method based on the original sampling waveform is close to that of the method in this paper, but the method has poor recognition performance for 16QAM and 64QAM. Similarly, the method based on the time-frequency map has a higher recognition rate for BPSK, 16QAM, and 64QAM but poor recognition performance for 2FSK, MSK, QPSK, and 2ASK. The method in this paper can effectively identify all kinds of modulation modes, and the recognition rate is more than 90%. This shows that the hybrid feature can effectively utilize the advantages of different features to improve the recognition rate.

**Figure 12:**
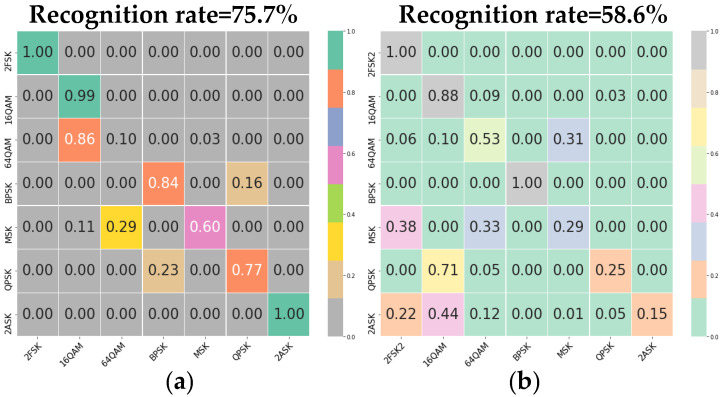
Experimental results on signal dataset 2 in which the signal parameters to be recognized are inconsistent with those of the training set. (**a**) Original sampling waveform. (**b**) Time-frequency map.

When the parameters of the training set and the test set are inconsistent, we use application dataset 2 to test the network structure and save training weights obtained through signal dataset 1; the test results are shown in Figure 12. It shows the confusion matrices obtained through 1D-CNN or 2D-CNN based on dataset 2 when we use one of the original sampling waveforms and time-frequency maps as input. The overall recognition rate of the method based on the original sampling waveform is 75.7%, and that of the method based on the time-frequency map is 58.6%. Both are lower than the method in this paper. Remarkably, the recognition rate based on the time-frequency map decreases significantly.

**Figure 13:**
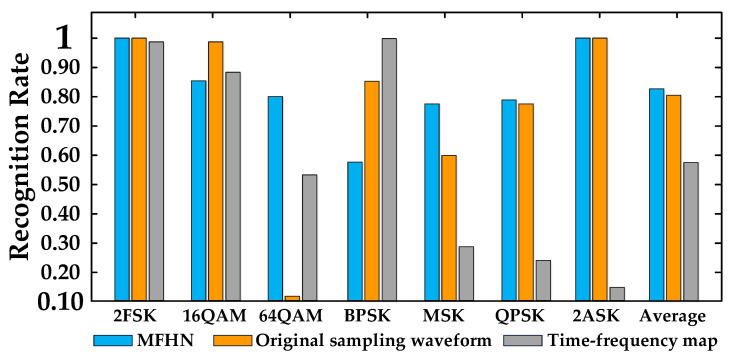
Modulation recognition based on signal dataset 2.

Figure 13 shows the recognition rate for a single modulation mode through MFHN (mixed features), 1D-CNN (original sampling waveforms), and 2D-CNN (signal time-frequency maps) based on dataset 2. When the parameters of the signal to be identified are changed, the recognition rate of 64QAM, 2FSK, MSK, QPSK, and 2ASK modulations by the method based on the original sampling waveform decreases significantly, and it is slightly improved for 16QAM. However, the recognition performance of the method using a time-frequency map as input decreases greatly. Although it has a high recognition rate for BPSK, the recognition rate for MSK, QPSK, and ASK is less than 30%, which seriously affects the practical application. It is shown that when the parameters are changed, the pattern recognition method relying on shape matching alone has poor applicability, and a large number of signal details are lost in time-frequency maps. Instead, there is no information loss in the original signal waveform, so feature extraction can be performed to achieve signal recognition. However, the recognition performance of the method in this paper also decreases; in particular, the recognition rates of BPSK and MSK are poor, but it is better than the other two methods.

### 5.3. Comprehensive Performance Comparison

**Figure 14:**
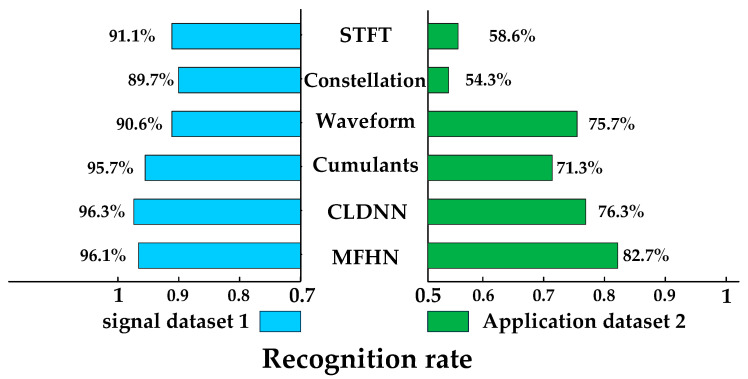
Comprehensive performance comparison with other methods, Results on dataset 1 mean that the signal parameters to be recognized are consistent with those of the training set. Results on dataset 1 mean that the signal parameters to be recognized are inconsistent with those of the training set.

Figure 14 shows the recognition rate for methods based on STFT, constellation, waveform, cumulant, CLDNN, and MFHN. To verify the practical application effect of our method, we use different datasets to compare our recognition rates with other methods. From Figure 14, it can be seen that when the parameters of the signal to be identified are consistent with that of the training set, the recognition rates of the six algorithms are approximately 90% and the recognition rates of the methods in this paper and CLDNN [24] are more than 96%. When the parameters of the signal to be identified are not consistent with the training set parameters, the recognition performance of the six algorithms decreases considerably, among which image-based methods such as the time-frequency map and constellation map are more affected. The cumulative method based on the original signal waveform is less affected, but the proposed method combines the advantages of different signal features and has a higher overall recognition rate than others, and therefore has a higher application value.

### 5.4. Recognition Result Based on Small Samples

**Figure 15:**
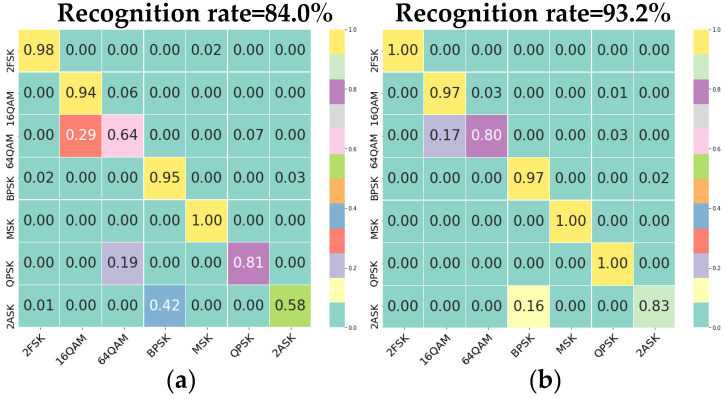
Experimental results on target dataset 3; the signal parameters to be recognized are inconsistent with those of the training set. (**a**) Recognition result of MFHN. (**b**) Recognition result of MJMN.

We analyze the performance of the MJMN framework. First, we test target signal dataset 3 using the MFHN trained from signal dataset 1; its result is shown in Figure 15a. Additionally, we use the trained model weights as the migration weights of the MJMN to train target dataset 3; the recognition results are shown in Figure 15b. The overall recognition rate and single modulation recognition rate of the latter are higher than those of the former, which verifies the validity of the MJMN.

In practice, we can obtain model weights as migration weights by training different types of modulation signals through an MFHN. When capturing new modulation signals with less data, we can use an MJMN to train it. As a result, new similar target signals can be quickly and accurately identified when they appear again.

## 6. Conclusions

To improve the recognition performance of real acquisition signals in wireless sensor networks, we design an RF database embedded in the signal preprocessing operators, improving the generalization ability of the neural network model. Furthermore, we propose a multidimensional feature hybrid network framework and a multifeatured joint migration network framework, which effectively improves the signal recognition performance, whose parameters are inconsistent with those of the training dataset. The proposed method improves the signal recognition performance of the single monitoring node and provides an effective reference for decision fusion in multi-sensor node modulation recognition. However, the recognition rate of the proposed network for unknown signals still needs to be improved. Next, we will carry out signal modulation recognition research from the direction of channel correction.

## Figures and Tables

**Table 1:** Delay and power distribution for multipath channels.

Path No.	Path Variances	Path Delays
1	0.865	50 ns
2	0.117	100 ns
3	0.016	150 ns
4	0.002	200 ns
5	0.0003	250 ns

**Table 2:** Database storage items.

	Storage Items	Generation Method	Storage Format
(a)	Sampling Signal File	Direct sampling	Records in database
(b)	Preprocessed Signal File	Signal precipitation and other operations on (a).	Records
(c)	Signal Dataset	Intercept sample points from (b)	Samples screened from records
(d)	Feature Dataset	Extract eigenvectors in units of one sample in (c)	Samples
(e)	Training Result	Recognition results and corresponding datasets	Records
(f)	Application Dataset	Generate in the same way with (c)	Samples
(g)	Application Feature Dataset	Generate in the same way with (d)	Samples
(h)	Application Result	Application recognition results and datasets	Records

**Table 3:** Database index items.

Source Attributes	Acquisition Parameters	Basic Signal Characteristics
Data Type	Data Format	Frequency Point
Source Coordinates	Acquisition Time	Bandwidth
Source Position	Acquisition Device	Chip Rate
Network Attributes	Acquisition Type	Signal To Noise Ratio

**Table 4:** DNN Parameters.

Layers	Kernel Sizes	Output Size
Input 1	—	8 × 1
LSTM 1	25	25 × 1
Dense 2 + ReLu	10	10 × 1
Dense 3 + ReLu	10	10 × 1
Dense 4 + ReLu	10	10 × 1
Dense 5 + ReLu	10	10 × 1

**Table 5:** 1D-CNN Parameters.

Layers	Kernel Sizes	Output Size
Input 2	—	512 × 1
Conv 7 + BN + Relu	256 × (1,3)	256 × 256 × 1
Conv 8 + BN + Relu	256 × (1,1)	256 × 128 × 1
Conv 9 + BN + Relu	80 × (3,3)	80 × 64 × 1
Conv 10 + BN + Relu	80 × (3,3)	80 × 32 × 1
Bi-LSTM 11	100	100 × 1
Dense 12 + Relu	50	50 × 1
Dense 13 + Relu	7	7 × 1

**Table 6:** 2D-CNN Parameters.

Layers	Kernel Sizes	Output Size
Input 3	—	128 × 128
Conv 14 + BN + Relu	64 × (3,3)	64 × 64 × 64
Conv 15 + BN + Relu	128 × (3,3)	32 × 32 × 128
Conv 16 + BN + Relu	256 × (3,3)	16 × 16 × 256
Dense 17 + Relu	65,536	65,536 × 1
Dense 18 + Relu	4096	4096 × 1
Dense 19 + Relu	1000	1000 × 1
Dense 20 + Relu	7	7 × 1

## Data Availability

Not applicable.
